# The Anatomy of the Regions Interested in Surgical Operations Performed upon the Human Body; with Occasional Views of the Pathological Conditions Which Render the Interference of the Surgeon Necessary. In a Series of Plates the Size of Life

**Published:** 1836-07

**Authors:** 


ART. VII.—*The Anatomy of the Regions interested in Surgical Operations performed upon the Human Body; with occasional Views of the Pathological Conditions which render the Interference of the Surgeon necessary. In a Series of Plates the size of life.* By J. LEBAUDY, M.D. Parts I. II. III.—*London*, 1835. Fol.

WE always feel a degree of apprehension upon the announcement of a system of anatomical plates. This mode of teaching the science of anatomy is, we suspect, as frequently the means of evil as of good. The capability of having the parts of study comfortably laid upon the table ready dissected on clean white paper—all the disagreeables and labours of the dissecting room escaped, offers temptations of no small force to the mind of many a "walker of the hospitals." The absolute impossibility of acquiring correct anatomical knowledge from plates, be they ever so good, is, one might suppose, so self-evident, that, did we not hear the contrary gravely asserted, not only by the uninformed public, but by those whose education ought to have taught them better, we might deem all observations of ours superfluous; but knowing, as we do, that it is by no means an uncommon thing for a student to trust very far too much to these helps, we feel it to be our duty to lift the friendly voice of warning. What is it that the surgeon finds essentially requisite in the practice of his profession?—An operation of any difficulty cannot be performed without an accurate knowledge not only of the course and position of the blood-vessels and nerves, but of the relative position, size, depth from the surface, and appearance not only of each organ in the vicinity, but of each structure and tissue. He should also be familiar with the appearance and *feel*, not only of the parts in one individual, but of the same parts as varied by disease or natural formation. Anatomy is as much a science of the touch as of the sight. How is the picture-taught surgeon to judge of the difference between the artery and the nerve? to obviate the possibility of his tying one for the other? How is he to distinguish and recognize the succession of parts brought into view at every stage of almost every operation of importance? The knowledge afforded even by the most practised eye is here often insufficient, without the assistance of that which the frequent habit of handling the dead body has procured. It is, however, the abuse of these helps which we deprecate, not their use. To the practitioner in the country they afford valuable assistance; they refresh the memory on points which time has effaced, and which he has no opportunity of reviving by actual examination; and this is almost the only use to which we could desire them to be applied, a use, we fear, far too low in the scale of importance for the expectations of their authors. The foregoing observations are of course not applicable to plates intended to illustrate pathology. The utility of these no one can dispute; but even here it would be well if the proper design and object of such representations were better understood and acted on. We should not then have mathematical figures cut out of a diseased organ which nothing but an implicit reliance upon the veracity of the author could have induced us to believe an approach to reality. Even the best efforts of the pencil can but feebly represent the infinite variety of appearance and of colouring which we meet with in disease: what then is the value of a more feeble attempt at its representation? It can only mislead in the exact ratio of its want of accuracy. A want of truth, or truth only half told, in a pathological plate, is in some respect more unpardonable than in anatomy, because the variety of natural structure is such that an anatomical figure can only pretend to represent a *manner* of formation, which may in many minor points be found to differ in almost every subject: and this being understood, we are not implicitly led astray by a slight error; we examine and dissect for ourselves, and obtain the general truth from the numberless particulars we meet with. A representation of disease, on the contrary, is a history of discovery, and every such history is not a mere *manner*, but a matter of fact, to a certain degree isolated and distinct from all others of the kind, and as such to be represented with the most scrupulous accuracy, inasmuch as it claims to be our pilot and guide in unravelling the intricacies of disease.

As a mere series of plates, we admire the execution of M. Lebaudy's work both for clearness and accuracy, and they would for this reason form valuable illustrations for a good and complete manual of operative surgery; but the extreme meagerness of the text is such as greatly to injure the value of the work, and to give the author the character rather of a book-maker than of a practical surgeon. To the advanced student the observations on each plate are useless, for want of completeness of description; and to the practitioner they are equally useless, from a similar meagerness in the practical part. We confess that we feel sorry to see such able specimens of art thrown away by being yoked to such an unequal match in the matter of the description. Should the author find, as we anticipate, that the public will not award to his labours the recompense which he no doubt looks for, let him be assured that, if the only valuable part of his present work, the plates, were incorporated into a standard system of operative surgery, (and we grieve to say that in our own language such a work at present does not exist,) he would confer a boon of great value on the profession, and place the labours of his scalpel and his pencil in the rank which they deserve to hold in surgical literature. The author has injudiciously added to the expense of his work by introducing among his plates two on pathological subjects, not at all connected with the rest, or with the object professed in the commencement of the work. They are beautiful delineations of the particular subjects which they represent, and are therefore worse than lost by being thus misplaced.

